# Refined Lord–Shulman Theory for 1D Response of Skin Tissue under Ramp-Type Heat

**DOI:** 10.3390/ma15186292

**Published:** 2022-09-10

**Authors:** Mohammed Sobhy, Ashraf M. Zenkour

**Affiliations:** 1 Department of Mathematics and Statistics, College of Science, King Faisal University, P.O. Box 400, Al-Ahsa 31982, Saudi Arabia; 2 Department of Mathematics, Faculty of Science, King Abdulaziz University, Jeddah 21589, Saudi Arabia; 3 Department of Mathematics, Faculty of Science, Kafrelsheikh University, Kafrelsheikh 33516, Egypt

**Keywords:** refined L–S model, bio-thermoelasticity, skin tissue, ramp-type heating

## Abstract

In this article, we present a mathematical model of thermoelastic skin tissue based on a refined Lord–Shulman heat conduction theory. A small thickness of skin tissue is considered to be one-dimensional with mechanical clamped surfaces. In addition, the skin tissue’s outer surface is subjected to ramp-type heating while its inner surface is adiabatic. A simple Lord–Shulman theory, as well as the classical coupled thermoelasticity, are also applied in this article. Laplace transform techniques and their inversions are calculated to return to the time domain. Numerical outcomes are represented graphically to discuss the significant impacts on the temperature, dilatation, displacement, and stress distributions. Such results provide a more comprehensive and better insight for understanding the behavior of skin tissue during the temperature distribution of a specific boundary condition.

## 1. Introduction

Different bioheat transfer investigations have become prominent in medicine due to the fast improvement of laser, microwave, radio-frequency, and focused ultrasound. Currently, such bioheat transfer representations can be effectively combined with experimental evidence on the human musculoskeletal and integumentary system thermal properties to provide various estimations of thermal characteristics, such as temperature, displacement, dilatation, and stress, as well as other thermal damages. Thus, experts can obtain knowledge about tissue and organ wounds and consumption systems, change current treatments, and foster novel devices for effective recovery of damaged tissues and organs. Bioheat transfer models can be used to cost-effectively evaluate the proficiency of treatments and can determine their risks. Creating models for analytic and treatment purposes can create virtual experiences in which the interactions are less tedious and provide important design information about the characteristics that should be changed while a product is still under improvement.

Various studies have evaluated the human musculoskeletal and integumentary scheme structures’ responses to directly utilized heat sources. A review on thermal extirpation of biological tissues in disease treatment has been presented by Singh and Melnik [1]. Another review on bioheat versions that were revised when employed in different hyperthermia treatments of cancer has been presented by Andreozzi et al. [2]. Fan et al. [3] investigated thermal and neural performances of skin tissue under thermal loads by establishing the thermal shock opposition of the skin. Khiavi et al. [4] solved the Pennes equation with respect to suitable thermal/physiological properties for each portion. Li et al. [5] presented the thermomechanical reply of porous biological tissue subjected to an immediate thermal shock in the text of a non-stability heat transfer pattern. Sur et al. [6] analyzed the bioheat relation in the text of remembrance replies and the three-phase-lag (TPL) theory under the two-temperature theory. Li et al. [7] submitted a semianalytical technique to explain the drawback of heat transfer in three-layered skin tissues and the expected temperature and thermal loss of skin tissues. Etehadtavakol and Ng [8] investigated the heat transfer and blood movement rate in biological procedures challenging perfect numerical patterns. Zhang and Shang [9] investigated 2D non-Fourier heat conduction in smooth skin tissue illuminated by a laser beam on its face based on bio-thermomechanics. Abdalla et al. [10] presented mathematical arrangements of the nonlinear bioheat hypothesis with energy dissipation to assess the temperature because of outer heat sources by employing certain dimension data on the biological tissue.

Fractional hyperbolic bioheat transfer (FBT) models have been used by several investigators. Kumar and Rai [11] created an FBT pattern by utilizing the fractional Taylor series formula for a single-phase-lag (SPL) formula. Ezzat [12] presented the FBT of the thermo-viscoelasticity model with varying thermal conductivity and rheological properties of the volume to examine bio-thermomechanics performance in living tissue. Hobiny et al. [13] used the FBT pattern as well as thermal relaxations on the temperature distribution of skin tissue and the ensuing thermal damage. Zhang et al. [14] used the FBT pattern and memory-dependent derivative as useful techniques to calculate the transient thermal replies with SPL and the resultant bioheat transfer relations. Wang et al. [15] developed a time-space FBT formula to examine the non-Fourier bioheat transfer procedure contained by the living biological tissues through laser irradiation.

Kumar and Rai [16] solved the perfect dual-phase-lag (DPL) pattern of bioheat transfer utilizing the finite element Legendre wavelet Galerkin approach. Kumar et al. [17] dealt with some information about the heat transfer in skin tissues utilizing a nonlinear DPL bioheat transfer pattern under regular heat flux boundary conditions. Sahoo et al. [18] investigated the internal temperature dissemination within tissue mimics implanted with and without gold nanostructures. They validated their experimental outcomes with the DPL model of Pennes bioheat conduction that incorporated a dual-phase lag model. Li et al. [19] derived the finite element control formulae of skin tissue in the context of generalized bio-thermoelasticity and obtained the nonlinear thermoelastic reactions of the three-layered skin tissue in the time domain immediately. Kumar et al. [20] presented a TPL model to define heat transfer in a finite domain skin tissue with temperature-dependent metabolic heat generation. Sharma and Kumar [21] studied the bioheat transfer under the Dirichlet boundary condition and due to complex nonlinear DPL bioheat transfer.

Kumar et al. [22] presented a nonlocal DPL model to adjust the impacts of thermo-mass and size-dependent thermophysical properties in a bilayer tissue at nanoscale heat transport. Li et al. [23] studied the coupling phase change heat transfer activity and thermal stress performance of living tissue through cryosurgery based upon a generalized thermoelastic theory. Hobiny and Abbas [24] determined a numerical study of thermal damage to living tissues utilizing a nonlinear DPL theory. Youssef and Alghamdi [25] composed and utilized bioheat transfer relations in the framework of a two-temperature heat conduction formula to examine the 3D disparity in the temperature of laser-irradiated living tissue. Youssef and Alghamdi [26] dealt with the temperature and the retort of skin tissue due to a uniform surface heat flux. Zhang et al. [27] presented the thermal reaction of skin tissue in the framework of a three-phase-lag (TPL) model of heat conduction. Chaudhary et al. [28] studied multilayer skin burn injuries employing the DPL bioheat model when the skin surface was exposed to various non-Fourier boundary conditions. Skin is considered to be a three-layered model known as the epidermis, dermis, and subcutaneous layer. Ma et al. [29] applied the DPL bioheat transfer pattern and Henrique’s burn assessment formula to define the response of multi-pulse heat sources and the skin. Zhang et al. [30] studied the thermoelastic reactions of living tissues exposed to an unexpected input of temperature loading in the framework of generalized thermoelasticity with the TPL model. Majchrzak and Stryczyński [31] considered a single blood vessel surrounded by biological tissue with a tumor based upon the DPL convention. Zhang et al. [32] analyzed the thermoelastic replies of skin tissue during laser irradiation in the framework of a generalized DPL pattern. Ghasemi et al. [33] used a DPL transient non-Fourier heat conduction in a functionally graded (FG) cylindrical material that is methodically resolved under axial heat flux conditions. Ezzat and Alabdulhadi [34] presented a unique mathematical pattern of generalized thermo-viscoelasticity theory based on Pennes bioheat transfer relation with DPL to examine the bio-thermomechanic responses in skin tissue. Ciesielski and Mochnacki [35] discussed a model of thermal procedures in the domain of living tissue secured by multilayered defensive clothing being in thermal contact with the environment. In addition, they presented a 2D thermomechanical reply of skin tissue exposed to various types of thermal loadings [36].

Modeling and knowledge of heat transport and temperature variations in living tissues and body organs are essential issues in medical thermal treatments. In this article, the refined L–S generalized thermoelastic theory has been applied to solve a ramp-type heating bio-thermoelastic coupling problem. The simple L–S and CPT models are also summarized. The aim is to solve such a problem of 1D and improve a new generalized bio-thermoelastic theory. The impacts of thermal relaxation and ramp-type heating parameters on the distributions across the skin tissue of temperature, displacement, dilatation, and stress are examined and represented graphically.

## 2. Governing Equations

The Fourier’s law that connects the heat flux vector, $\vec{q}$, to the temperature gradient, ∇θ, is given here [25,26,27,28]:

(1) $$\vec{q}\left(\vec{x},t\right)=-k_{t}\nabla \theta \left(\vec{x},t\right),$$

where $k_{t}$ implies the thermal conductivity coefficient of the tissue and $\theta =T-T_{b}$ denotes the absolute temperature of the tissue, in which $T_{b}$ denotes the arterial blood temperature. The energy balance equation is expressed as [25,26,27,28]:

(2) $$\rho_{t}c_{t}\frac{\partial \theta }{\partial t}+\gamma_{t}T_{0}\frac{\partial }{\partial t}\left(\mathrm{div}\vec{u}\right)=-\nabla \cdot \vec{q}+Q_{b}+Q_{m}+Q_{r},$$

where $c_{t}$ denotes the heat capacity of a unit mass of the tissue, $\rho_{t}$ represents the mass density of the tissue, $\vec{u}$ represents the displacement vector and $\mathrm{div}\vec{u}=e=e_{kk}$ denotes volumetric strain, $e_{ij}$ represents strain tensor, and $\gamma_{t}$ signifies the thermal modulus which is given in terms of the thermal expansion coefficient $\alpha_{t}$ as $\gamma_{t}=\left(2\mu_{t}+3\lambda_{t}\right)\alpha_{t}$ in which $\lambda_{t}$ and $\mu_{t}$ are Lamé constants of the tissue. For biological tissue, $Q_{b}$, $Q_{m}$, and $Q_{r}$ represent the perfusion heat source, the metabolic hotness source, and the remotely applied heat source term, respectively. The metabolic heat hotness is delivered in the body by the admission of food, and the perfusion heat rate is the hotness source that is spent in blood flow. The blood perfusion heat source $Q_{b}$ shows convection in the blood. It eliminates heat because of the flow of blood and has been described by numerous analysts as [25,26,27,28]:

(3) $$Q_{b}=w_{b}\rho_{b}c_{b}\left(T_{b}-T\right),$$

where $w_{b}$ represents the perfusion rate of arterial blood; $\rho_{b}$ and $c_{b}$ denote the specific heat and mass density of the blood, respectively.

Albeit the old-style thermoelasticity (CTE) hypothesis has been utilized in manufacturing for its straightforward structure and overall powerful arrangement, the coupled theory based on Fourier’s conduction law has been improved to represent non-Fourier peculiarity in certain media, auch as sand and living tissue. To encourage the PL trend that followed in the bioheat transfer procedure, a relaxation time τ was established for the heat conduction. Fourier law is replaced by the Cattaneo–Vernotte model of thermal conductivity that includes both heat flow and its time derivatives and a relaxation time τ, and is given by [37,38,39,40,41,42,43]:

(4) $$\left(1+\sum_{n=1}^{N}\frac{\tau^{n}}{n!}\frac{\partial^{n}}{\partial t^{n}}\right)\vec{q}=-k_{t}\nabla \theta .$$

In sequence with the energy conservative law (2) with Equation (4), we obtain the modified heat conduction relation with the first relaxation time τ as [44,45,46,47,48]:

(5) $$k_{t}\nabla^{2}\theta =\left(1+\sum_{n=1}^{N}\frac{\tau^{n}}{n!}\frac{\partial^{n}}{\partial t^{n}}\right)\left[\frac{\partial }{\partial t}\left(\rho_{t}c_{t}\theta +\gamma_{t}T_{b}e\right)+w_{b}\rho_{b}c_{b}\theta -Q_{m}-Q_{r}\right],\;N\geq 1,$$

which denotes the refined L–S generalized thermoelasticity theory. The simple L–S generalized thermoelasticity theory is achieved when N=1 in the form:

(6) $$k_{t}\nabla^{2}\theta =\left(1+\tau \frac{\partial }{\partial t}\right)\left[\frac{\partial }{\partial t}\left(\rho_{t}c_{t}\theta +\gamma_{t}T_{b}e\right)+w_{b}\rho_{b}c_{b}\theta -Q_{m}-Q_{r}\right],$$

while the CTE theory is obtained by setting τ=0 as:

(7) $$k_{t}\nabla^{2}\theta =\frac{\partial }{\partial t}\left(\rho_{t}c_{t}\theta +\gamma_{t}T_{b}e\right)+w_{b}\rho_{b}c_{b}\theta -Q_{m}-Q_{r}.$$

It is to be noted that the classical coupled thermoelasticity theory suggested by Biot [10] firstly contains the above energy conservative equation, as well as the forthcoming equation of motion.

For the present 1D problem, the constitutive relations can be reduced to:

(8) $$\sigma =\left(\lambda_{t}+2\mu_{t}\right)e-\gamma_{t}\theta ,$$

where

(9) $$e=\frac{\partial u}{\partial x},$$

and the equations of motion without considering the body force can be reduced to:

(10) $$\left(\lambda_{t}+2\mu_{t}\right)\frac{\partial^{2}u}{\partial x^{2}}-\gamma_{t}\frac{\partial \theta }{\partial x}=\rho_{t}\frac{\partial^{2}u}{\partial t^{2}}.$$

The above equation of motion itself is used with Equation (7) to formulate the CTE model, with Equation (6) to form the simple L–S model, and finally with Equation (5) to formulate the refined L–S model.

## 3. Mathematical Solution to the Problem

Considering the refined L–S model, Equations (5), (8) and (10), with neglecting $Q_{r}$, can be expressed as:

(11) $$\frac{\partial^{2}u}{\partial x^{2}}-c_{1}\frac{\partial \theta }{\partial x}=\frac{1}{C_{P}^{2}}\frac{\partial^{2}u}{\partial t^{2}},$$

(12) $$C_{T}^{2}\frac{\partial^{2}\theta }{\partial x^{2}}=\left(1+\sum_{n=1}^{N}\frac{\tau^{n}}{n!}\frac{\partial^{n}}{\partial t^{n}}\right)\left[\left(w_{b}\rho_{c}+\frac{\partial }{\partial t}\right)\theta +\eta \frac{\partial^{2}u}{\partial t\partial x}\right]-Q_{0},$$

(13) $$\frac{\sigma }{\lambda_{t}+2\mu_{t}}=\frac{\partial u}{\partial x}-c_{1}\theta ,$$

where

(14) $$c_{1}=\frac{\gamma_{t}}{\lambda_{t}+2\mu_{t}},\;\;C_{P}^{2}=\frac{\lambda_{t}+2\mu_{t}}{\rho_{t}},\;\;\;C_{T}^{2}=\frac{k_{t}}{\rho_{t}c_{t}},\;\;\;\rho_{c}=\frac{\rho_{b}c_{b}}{\rho_{t}c_{t}},\;\;\;\eta =\frac{\gamma_{t}T_{b}}{\rho_{t}c_{t}},\;\;\;Q_{0}=\frac{Q_{m}}{\rho_{t}c_{t}}.$$

Now, we present the initial and boundary conditions of the problem. The initial conditions of the considered issue are thought to be homogeneous as follows:

(15) $${u\left(x,t\right)|}_{t=0}={\frac{\partial^{n}u\left(x,t\right)}{\partial t^{n}}|}_{t=0}=0,\;\;\;{\theta \left(x,t\right)|}_{t=0}={\frac{\partial^{n}\theta \left(x,t\right)}{\partial t^{n}}|}_{t=0}=0,\;\;\;\;\;n\geq 1.$$

**Figure 1:**
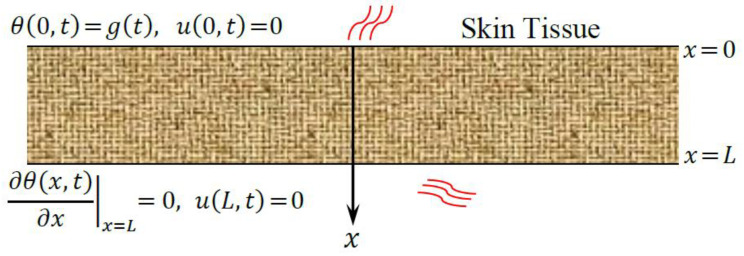
The model of skin tissue with boundary conditions.

The biological tissue is believed to be fixed on both outer and inner surfaces. Thermal loading is utilized on the upper surface of the skin tissue, while its inner surface remains at the rated temperature vanishing. Therefore, the boundary conditions are stated as:

(16) $$\theta \left(0,t\right)=g\left(t\right),\;\;\;\;{\frac{\partial \theta \left(x,t\right)}{\partial x}|}_{x=L}=0,\;\;\;\;\;u\left(0,t\right)=0,\;\;\;\;\;u\left(L,t\right)=0,$$

where g(t) denotes the thermal load function on the upper surface of the skin tissue x=0, as shown in Figure 1. Second, we assume that the plane x=0 of the tissue is subjected to ramp-type heating as follows:

(17) $$g\left(t\right)=\theta_{0}\{\begin{array}{c} \frac{t}{t_{0}}\;\;\;\;\;\mathrm{if}\;\;\;\;\;0<t<t_{0},\\ 1\;\;\;\;\;\;\;\;\mathrm{if}\;\;\;\;\;\;\;\;\;t\geq t_{0}, \end{array}$$

where $\theta_{0}>0$ is a constant that represents the thermal loading and $t_{0}>0$ is the ramp-type heating parameter.

## 4. Laplace Transform Domain and Its Inversion

Taking the Laplace transform characterized by the connection:

(18) $$\overset{-}{f}\left(x,s\right)=\int_{0}^{\infty }e^{-st}f\left(x,t\right)dt,$$

to the two sides of Equations (11)–(13) and utilizing homogeneous initial conditions (15), we gain field equations in the Laplace change space as follows:

(19) $$\left(\frac{d^{2}}{dx^{2}}-c_{2}\right)\bar{u}-c_{1}\frac{d\bar{\theta }}{dx}=0,$$

(20) $$\left(\frac{d^{2}}{dx^{2}}-c_{3}\right)\bar{\theta }=c_{4}\frac{d\bar{u}}{dx}-{\bar{Q}}_{1},$$

(21) $$\frac{\bar{\sigma }}{\lambda_{t}+2\mu_{t}}=\frac{d\bar{u}}{dx}-c_{1}\bar{\theta },$$

where

(22) $$c_{2}=\frac{s^{2}}{C_{P}^{2}},\;\;\;c_{3}=\frac{w_{b}\rho_{c}+s}{C_{T}^{2}}\left(1+\sum_{n=1}^{N}\frac{\tau^{n}}{n!}s^{n}\right),\;c_{4}=\frac{\eta s}{C_{T}^{2}}\left(1+\sum_{n=1}^{N}\frac{\tau^{n}}{n!}s^{n}\right),\;\;Q_{1}=\frac{Q_{0}}{C_{T}^{2}}.$$

It is noted that the over bar image represents its Laplace transform and s indicates the Laplace parameter. The system of equations that appear in Equations (19) and (20) is solved in the Laplace domain to obtain:

(23) $$\bar{\theta }=\sum_{i=1}^{2}\left(A_{i}\;e^{\xi_{i}x}+B_{i}\;e^{-\xi_{i}x}\right)+{\bar{Q}}_{2},$$

(24) $$\bar{u}=\sum_{i=1}^{2}\beta_{i}\left(A_{i}\;e^{\xi_{i}x}-B_{i}\;e^{-\xi_{i}x}\right),$$

where $A_{i}$ and $B_{i}$ are constant coefficients differing on s and ${\bar{Q}}_{2}={\bar{Q}}_{1}/c_{3}$. The parameters $\xi_{i}$ and $\beta_{i}$ are given by:

(25) $$\xi_{1},\xi_{2}=\frac{1}{\sqrt{2}}\sqrt{c_{1}c_{4}+c_{2}+c_{3}\pm \xi_{0}},\;\;\xi_{0}=\sqrt{{\left(c_{1}c_{4}+c_{2}\right)}^{2}+c_{3}\left[c_{3}+2\left(c_{1}c_{4}-c_{2}\right)\right]},$$

(26) $$\beta_{i}=\frac{\xi_{i}\left(\xi_{i}^{2}-c_{1}c_{4}-c_{3}\right)}{c_{2}c_{4}}.$$

In addition, the dilatation in Equation (9) is given in the Laplace domain by:

(27) $$\bar{e}=\sum_{i=1}^{2}\beta_{i}\xi_{i}\left(A_{i}\;e^{\xi_{i}x}+B_{i}\;e^{-\xi_{i}x}\right).$$

Additionally, the axial stress according to Equation (21) becomes:

(28) $$\bar{\sigma }=\sum_{i=1}^{2}\zeta_{i}\left(A_{i}\;e^{\xi_{i}x}+B_{i}\;e^{-\xi_{i}x}\right)-{\bar{Q}}_{3},$$

where

(29) $$\zeta_{i}=\left(\lambda_{t}+2\mu_{t}\right)\left(\beta_{i}\xi_{i}-c_{1}\right),\;\;\;\;\;{\bar{Q}}_{3}=\left(\lambda_{t}+2\mu_{t}\right)c_{1}{\bar{Q}}_{2}.$$

In the Laplace transform domain, the boundary conditions (16) are given by:

(30) $${\overset{-}{\theta }\left(x,s\right)|}_{x=0}=\frac{\theta_{0}\left(1-e^{-t_{0}s}\right)}{t_{0}s^{2}}={\overset{-}{G}}_{s},$$

(31) $${\frac{\partial \overset{-}{\theta }\left(x,s\right)}{\partial x}|}_{x=L}=0,\;\;\;\;{\overset{-}{u}\left(x,s\right)|}_{x=0,L}=0.$$

The solution of the exceeding arrangement of direct conditions provides the obscure parameters $A_{i}$ and $B_{i}$. Applying the above boundary conditions on Equations (23) and (24), hence one obtains:

(32) $$\left[\begin{array}{cccc} 1 & 1 & 1 & 1\\ \xi_{1}e^{\xi_{1}L} & -\xi_{1}e^{-\xi_{1}L} & \xi_{2}e^{\xi_{2}L} & -\xi_{2}e^{-\xi_{2}L}\\ \beta_{1} & -\beta_{1} & \beta_{2} & -\beta_{2}\\ \beta_{1}e^{\xi_{1}L} & -\beta_{1}e^{-\xi_{1}L} & \beta_{2}e^{\xi_{2}L} & -\beta_{2}e^{-\xi_{2}L} \end{array}\right]\left\{\begin{array}{c} \begin{array}{c} A_{1}\\ B_{1} \end{array}\\ \begin{array}{c} A_{2}\\ B_{2} \end{array} \end{array}\right\}=\left\{\begin{array}{c} \begin{array}{c} {\overset{-}{G}}_{s}-{\bar{Q}}_{2}\\ 0 \end{array}\\ \begin{array}{c} 0\\ 0 \end{array} \end{array}\right\}.$$

To achieve the solutions in the Laplace transform domain, we solve the above system of linear equations to obtain the following parameters:

(33) $$A_{1}=\frac{1}{\Delta }\beta_{2}\left({\overset{-}{Q}}_{2}-{\overset{-}{G}}_{s}\right)\left(e^{\left(\xi_{1}-\xi_{2}\right)L}-e^{\left(\xi_{1}+\xi_{2}\right)L}\right),$$

(34) $$B_{1}=\frac{1}{\Delta }\beta_{2}\left({\overset{-}{Q}}_{2}-{\overset{-}{G}}_{s}\right)\left(e^{\left(3\xi_{1}-\xi_{2}\right)L}-e^{\left(3\xi_{1}+\xi_{2}\right)L}\right),$$

(35) $$A_{2}=\frac{1}{\Delta }\beta_{1}\left({\overset{-}{G}}_{s}-{\overset{-}{Q}}_{2}\right)\left(e^{\left(\xi_{1}-\xi_{2}\right)L}-e^{\left(3\xi_{1}-\xi_{2}\right)L}\right),$$

(36) $$B_{2}=\frac{1}{\Delta }\beta_{1}\left({\overset{-}{G}}_{s}-{\overset{-}{Q}}_{2}\right)\left(e^{\left(\xi_{1}+\xi_{2}\right)L}-e^{\left(3\xi_{1}+\xi_{2}\right)L}\right),$$

in which

(37) $$\Delta =\left(\beta_{1}+\beta_{2}\right)\left(e^{\left(\xi_{1}+\xi_{2}\right)L}-e^{\left(3\xi_{1}-\xi_{2}\right)L}\right)+\left(\beta_{1}-\beta_{2}\right)\left(e^{\left(\xi_{1}-\xi_{2}\right)L}-e^{\left(3\xi_{1}+\xi_{2}\right)L}\right).$$

This finishes the solution to the problem in the transform domain. Since the expressions in Equations (23) and (24) are extremely difficult, it is extremely complicated to achieve the inverse transform in an analytical form in the time domain. Hence, the numerical inverse Laplace transform method is utilized to achieve the reactions of temperature, displacement, dilatation, and stress in the actual time domain. To achieve numerical results in the physical domain, we employ the Riemann sum approximation technique. In this strategy, any function $\overset{-}{f}\left(x,s\right)$ in the space of Laplace transform is upset to the physical domain f(x,t) by applying the notable equation [49]:

(38) f(x,t)=eϱtt[12Re{f-(x,ϱ)}+Re{∑n=0N(f-(x,ϱ+inπt)(−1)n)}],

where Re is the real part of a function, $i=\sqrt{-1}$ and ϱ≈4.7/t [50].

## 5. Numerical Results

The numerical results concerning temperature, displacement, dilatation, and stress across the skin tissue are discussed here. The effect of the ramp-type heating parameter, the lack and existence of couple stress, and thermal relaxation time on field variables are examined. The thermophysical properties of the biological tissue and blood can be expressed as:

$$\begin{array}{c} \lambda_{t}=8.27\times 10^{8}\;\mathrm{kg}/\left({m\;s}^{2}\right),\;\;\;\;\mu_{t}=3.446\times 10^{7}\;\mathrm{kg}/\left({m\;s}^{2}\right);\\ \rho_{t}=1190\;\mathrm{kg}/m^{3},\;\;\;\;T_{b}=310\;K,\;\;\;\;c_{t}=3600\;J/\left(K\;\mathrm{kg}\right),\;\;\;\;k_{t}=0.235\;W/\left(m\;K\right);\\ \rho_{b}=1060\;\mathrm{kg}/m^{3},\;\;\;\;c_{b}=3770\;J/\left(K\;\mathrm{kg}\right),\;\;\;\;\alpha_{t}=1\times 10^{-4}\;\left(1/K\right). \end{array}$$

The thickness of the skin tissue is assumed to be L=1 mm and the metabolic heat source $Q_{m}=368.1\;W/m^{3}$. The values of temperature change (θ), distributions of displacement (u), dilatation (e), and stress (σ) are defined according to Equation (38). Numerical outcomes have been carried out and exhibited in detail in Figure 2, Figure 3, Figure 4, Figure 5, Figure 6, Figure 7, Figure 8, Figure 9, Figure 10, Figure 11 and Figure 12.

### 5.1. Refined, Simple, and Classical Models

**Figure 2:**
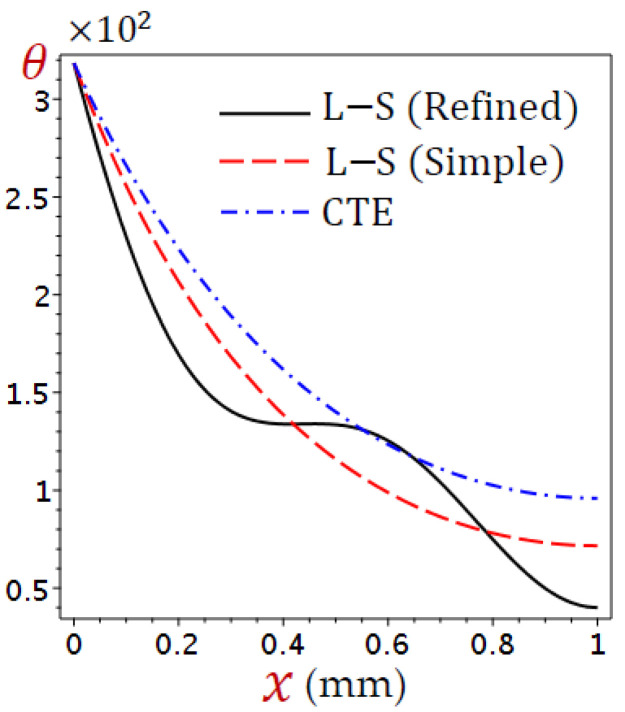
Distributions of temperature (θ) through the skin tissue using various theories.

**Figure 3:**
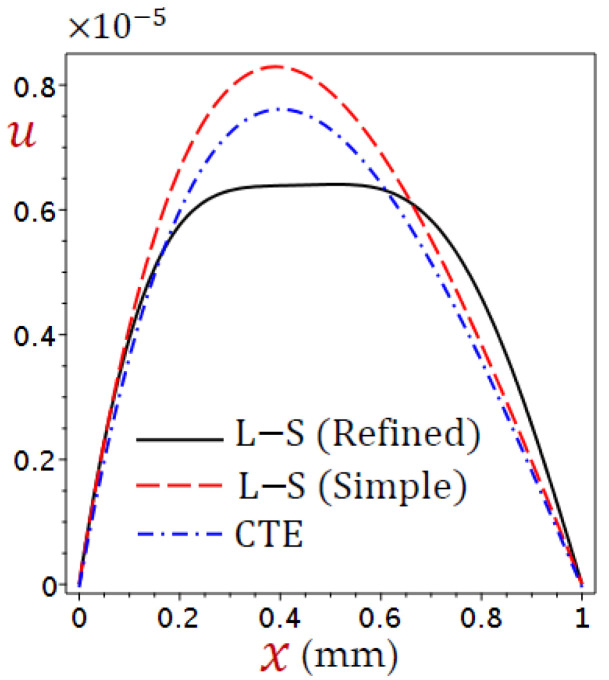
Distributions of displacement (u) through the skin tissue using various theories.

**Figure 4:**
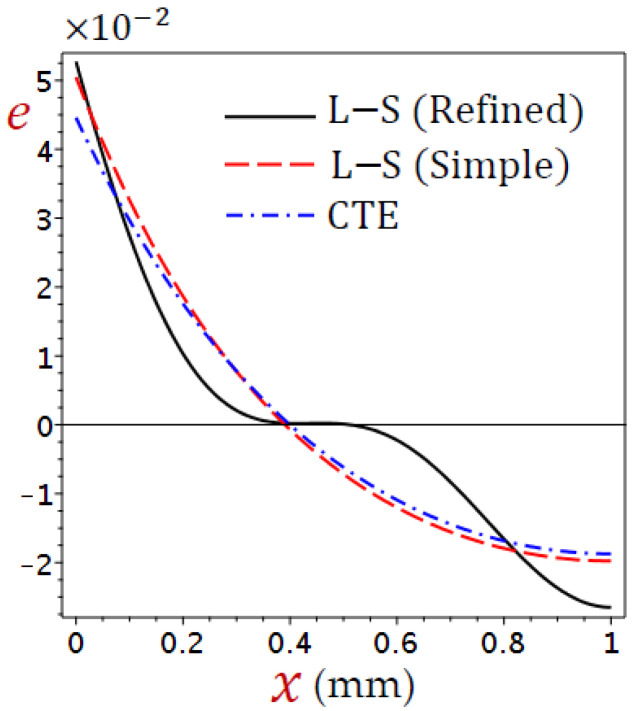
Distributions of dilatation (e) through the skin tissue using various theories.

**Figure 5:**
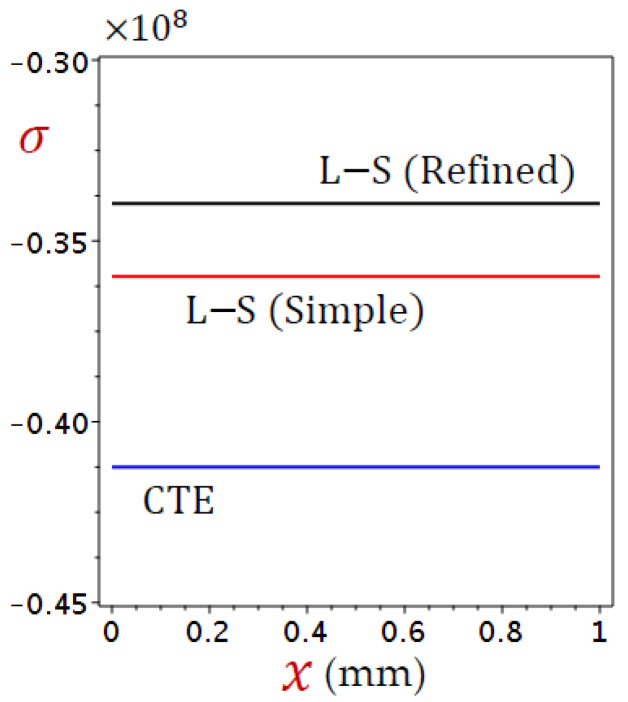
Distributions of stress (σ) through the skin tissue using various theories.

The field quantities due to different coupling theorems are illustrated in Figure 2, Figure 3, Figure 4 and Figure 5. Figure 2 displays the distributions of temperature (θ) through the skin tissue using various theories. The temperature decreases directly as x increases for the CTE and simple L–S generalized thermoelastic models. The behavior of θ due to the refined L–S generalized thermoelastic model has a different look. The temperature θ vibrates along with the thickness of the skin tissue.

Figure 3 displays the distributions of displacement (u) through the skin tissue using different theories. The displacement is no longer growing and has its maximum value at x=0.4 for the CTE and simple L–S generalized thermoelastic models. The simple L–S generalized thermoelastic model gives the largest displacement. Once again, the behavior of u due to the refined L–S generalized thermoelastic model has a different look. The maximum value of u occurs at x=0.6.

Figure 4 displays the distributions of dilatation (e) through the skin tissue using different theories. The dilatation decreases directly as x increases for the CTE and simple L–S generalized thermoelastic models. The behavior of e due to the refined L–S generalized thermoelastic model has a different look. The dilatation (e) vibrates along with the thickness of the skin tissue. It is interesting to see that the dilatation (e) vanishes when x=0.4 for all theories.

Figure 5 displays the distributions of stress (σ) through the skin tissue using different theories. It is clear that the stress (σ) is independent of x. This is due to the used boundary conditions. The refined L–S generalized thermoelastic model gives the largest stresses while the CTE provides the lowest ones.

### 5.2. The Impact of Ramp-Type Heating

**Figure 6:**
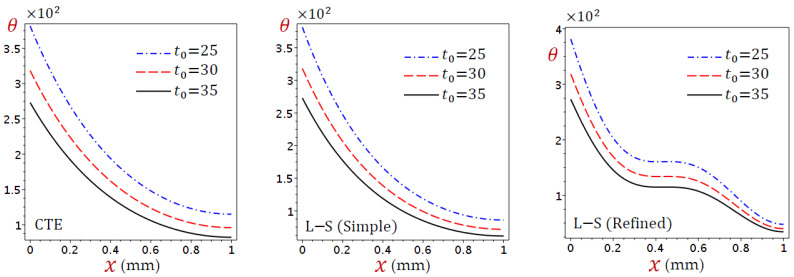
Distributions of temperature (θ) through the skin tissue according to various values of the ramp-type heating parameter.

**Figure 7:**
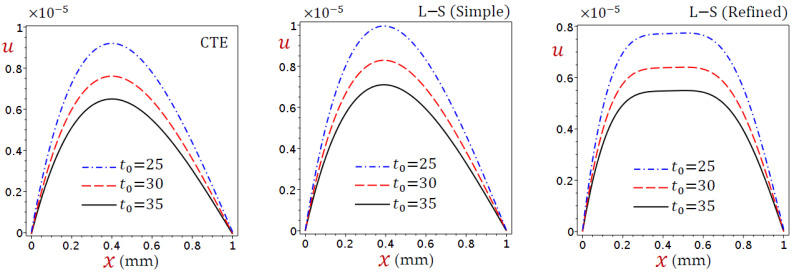
Distributions of displacement (u) through the skin tissue according to various values of the ramp-type heating parameter.

**Figure 8:**
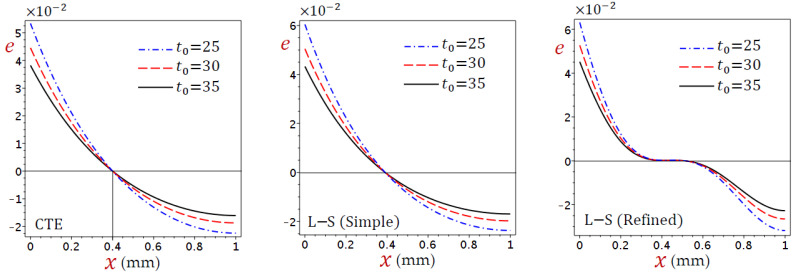
Distributions of dilatation (e) through the skin tissue according to various values of the ramp-type heating parameter.

As a second case, we consider the effects of the ramp-type heating parameter ($t_{0}$) on the temperature, displacement, and dilatation in Figure 6, Figure 7 and Figure 8. Different values of the ramp-type heating parameter are used in these figures. Once again, Figure 6 indicates that the distribution of temperature (θ) directly decreases through the skin tissue for the CTE and simple L–S generalized thermoelastic models. In addition, the behavior of θ due to the refined L–S generalized thermoelastic model has a different appearance; in general, θ decreases as $t_{0}$ increases.

In Figure 7, the displacement (u) vanishes at the skin tissue edges and this is due to the boundary conditions. In addition, its maximum value occurs when x=0.4 for the CTE and simple L–S generalized thermoelastic models. This is not the same as the refined L–S generalized thermoelastic model. In general, the displacement (u) decreases as $t_{0}$ increases.

The dilatation (e) directly decreases through the skin tissue, as shown in Figure 8, as a result of the CTE and simple L–S generalized thermoelastic models. It looks slightly different from the refined L–S generalized thermoelastic model. Once again, the dilatation (e) vanishes when x=0.4. For all theories, e decreases as the ramp-type heating parameter $t_{0}$ increases when x<0.4 and vice versa when x>0.4.

### 5.3. The Impact of the Thermal Relaxation Time

**Figure 9:**
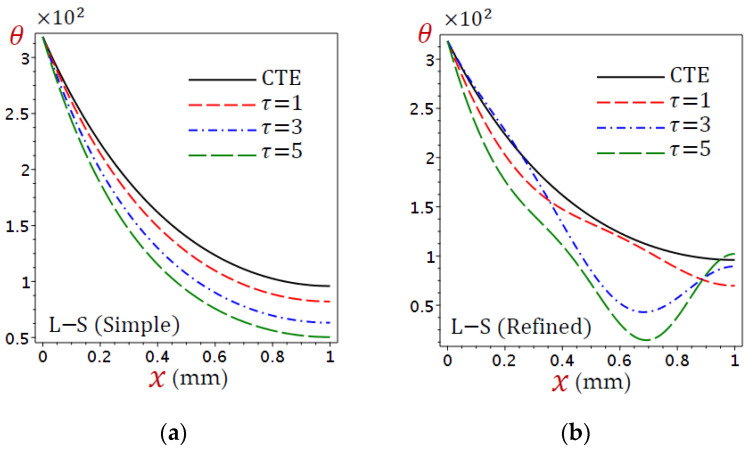
Distributions of temperature (θ) through the skin tissue according to various values of the relaxation time: (**a**) L–S (simple) and (**b**) L–S (refined).

**Figure 10:**
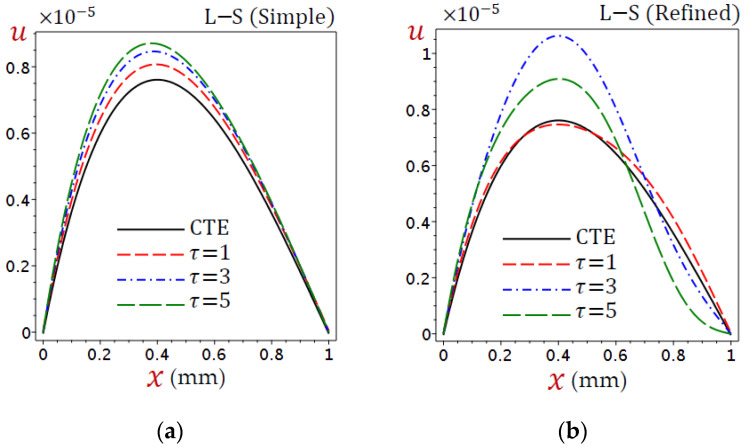
Distributions of displacement (u) through the skin tissue according to various values of the relaxation time: (**a**) L–S (simple) and (**b**) L–S (refined).

**Figure 11:**
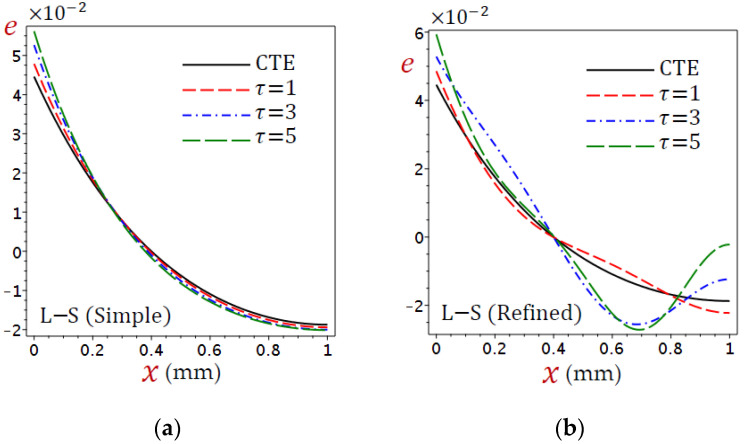
Distributions of dilatation (e) through the skin tissue according to various values of the relaxation time: (**a**) L–S (simple) and (**b**) L–S (refined).

In the last case, we consider an alternate estimation of thermal relaxation time (τ) when the ramp-type heating parameter remains constant ($t_{0}=0.3$). The graphs in Figure 9, Figure 10 and Figure 11 speak to the curves anticipated by the two unique theories of thermoelasticity obtained as exceptional instances of the current simple and refined L–S models. The simple and refined results are compared with those due to the CTE theory (τ=0). 

In Figure 9a, the temperature (θ) decreases directly across the thickness of skin tissue for the CTE and simple L–S generalized thermoelastic models. The distribution of temperature (θ) decreases as the relaxation time (τ) increases. Of these, the CTE theory provides the largest temperature. The behavior of θ is different in the case of the refined model (Figure 9b); θ has different shapes according to the values of τ.

It is clear in Figure 10a that the displacement (u) is uniformly increasing as the relaxation time (τ) increases for the simple L–S generalized thermoelastic model. The variation of τ has different non-uniformly effects on the displacement u for the refined L–S generalized thermoelastic model, as shown in Figure 10b.

For the simple L–S generalized thermoelastic model, Figure 11a illustrates that the dilatation (e) is uniformly increasing as the relaxation time (τ) increases when x<0.256 and vice versa when x>0.256. It is obvious that the variation of τ does not affect the dilatation when x=0.256 in which $e=1.15\times 10^{-2}$. Once again, the variation of τ has different non-uniformly effects on the dilatation e for the refined L–S generalized thermoelastic model, as shown in Figure 11b. The variation of τ does not affect the dilatation when x=0.4 in which e=0. The variation of τ also has similar effects on the dilatation e when x=0.84.

**Figure 12:**
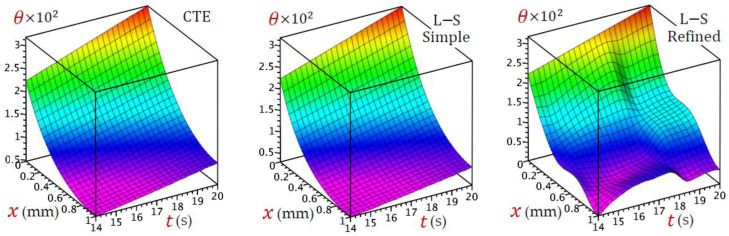
3D distributions of temperature (θ) versus time and across the skin tissue according to various theories.

For the sake of completeness, one additional figure is given to show the 3D distributions of temperature θ(x,t) versus time across the skin tissue according to different theories. It is noticed in Figure 12 that time (t) greatly affects the distribution of the temperature θ(x,t), especially, in the instance of the refined L–S generalized thermoelastic model.

## 6. Conclusions

In the current study, we consider the newly developed model of thermoelasticity based on a single delay term to be announced into the thermal conduction equation through the thickness of skin tissue. The system of the governing equations of the proposed model has been developed based on generalized thermoelasticity theory. Solutions to the physical fields of the skin tissue are obtained by applying the Laplace technique. The numerical technique have also been used to obtain solutions to different fields of the skin tissue in the physical field. The analytical and numerical studies of governing equations both show a significant influence on the ramp-type heating, relaxation time, and time parameters. This investigation is necessary for skin tissue problems because, in these cases, material parameters rely upon temperature. Finally, the current model may be used as a piece of bioheat transfer applications.

## Data Availability

Not applicable.
